# ‘*Unable to dodge the bullet’*: a qualitative study of ethical dilemmas and moral distress of critical care nurses during the Covid-19 pandemic in a South African Province

**DOI:** 10.1186/s12912-025-03405-1

**Published:** 2025-07-01

**Authors:** Busisiwe Sibiya, Laetitia C. Rispel

**Affiliations:** Centre for Health Policy & South African Research Chairs Initiative, School of Public Health, Faculty of Health Sciences, University of the Witwatersrand, Johannesburg, South Africa

**Keywords:** Ethical dilemmas, Moral distress, Covid-19, Nurses and critical/Intensive care

## Abstract

**Background:**

Worldwide, nurses have been at the frontline of the Covid-19 pandemic response and central to its effectiveness. They faced numerous ethical dilemmas which in turn resulted in considerable moral distress. However, there are knowledge gaps on the experiences of critical care nurses in South Africa during the pandemic.

**Aim:**

Explore the experiences, specifically the ethical dilemmas and moral distress, of critical care nurses working in South African hospitals.

**Methods:**

Gilligan’s ethic of care theory informed this exploratory, qualitative descriptive study with nurses who had experience of taking care of individuals with Covid-19 and working in intensive (critical) care units in the Gauteng province of South Africa. We recruited eligible nurses through a combination of social media adverts, snowballing, and referral from professional associations or trade unions. Following voluntary informed consent, we conducted in-depth interviews with nurses using an interview guide that focused on personal and professional experiences during the pandemic, ethical dilemmas, relationships with other colleagues and/or management, and the availability of support systems. Data was analysed thematically.

**Results:**

The participants comprised 21 nurses, 16 females and 5 males with a mean age of 38 years. The majority were professional nurses (20/21 = 95%) and from the public health sector (17/21 = 81%). Nurses highlighted the tension between their deep caring for patients and the realities of taking care of patients during the Covid-19 pandemic that necessitated pragmatic compromises, such as doing the bare minimum. They expressed ambivalence about the Nursing Oath because they were acutely aware of the moral obligation to put the health of their patients as their first consideration, yet they faced the personal risk of infections and disease exposure. The uncertainty and fear of the pandemic, of infection, of the unknown, and of being in the frontline of health care provision resulted in considerable moral distress. Simultaneously, the perceived lack of appreciation for their work and for risking their lives as health care providers, and the resource constraints intersected with and exacerbated both ethical dilemmas and moral distress.

**Conclusions:**

This study highlights the ethical dilemmas that nurses experienced during the pandemic, and their perceived moral distress. It contributes to the discourse on healthcare ethics, particularly in crisis situations, and highlights the need for robust support systems for nurses.

**Supplementary Information:**

The online version contains supplementary material available at 10.1186/s12912-025-03405-1.

## Background

The Covid-19 pandemic has underscored the chronic underinvestment in the health workforce and amplified the global crisis, characterised by shortages, migration, and inequitable distribution [1]. Worldwide, nurses have been at the frontline of the Covid-19 response and central to its effectiveness [2]. However, the pandemic negatively impacted nurses at both professional and personal levels [3]. Nurses were faced with large numbers of sick patients, increasing workloads, re-deployment to unfamiliar areas, new responsibilities, resource constraints, and demands to “cope” and be “resilient” [2]. They were challenged by the risk of personal infections, the difficulty of protecting themselves, exacerbated by inadequate access to personal protective equipment, isolation from family and friends, and stigma [4–6]. The pandemic also affected their physical and psychological well-being, which in turn influenced their ability to cope with the Covid-19 health crisis [7].

Critical care nurses were in the proverbial eye of the storm, due to the complex and demanding care provided to seriously ill patients and the gravity of their responsibilities in high-pressure environments [8]. They faced numerous ethical dilemmas during the Covid-19 pandemic, including difficult decisions regarding patient treatment options, resource allocation in the face of supplies or staff shortages, and balancing nursing care and their safety amidst personal protective equipment (PPE) shortages or inadequacies [2, 3, 5, 9]. These ethical dilemmas in turn resulted in considerable moral distress [3, 8, 10, 11]. Moral distress is defined as “a *stress that health professionals may experience when various obstacles prevent them from performing what they believe is ethically correct*” [8] p.614. Silverman highlights that the gap between ethical conviction and the inability to do the right thing leads to the experience of moral distress [12]. A systematic review that covered the period from 2012 to 2022 found that gender, futile and end-of-life care, fear of contracting and spreading Covid-19, decision-making about treatment processes, and poor teamwork contributed to moral distress among critical care nurses [8]. Another study in the Netherlands found that an increased patient load and working with inadequately ICU trained staff resulted in moral distress for nurses [11].

South Africa was disproportionately affected by the Covid-19 pandemic [13], which aggravated both the health system fragilities and the chronic health workforce crisis [14]. By the end of the pandemic, more than a 1 000 health workers had died of Covid-19 infections, the majority of whom were nurses [15]. As elsewhere, nurses make up the largest single group of health care providers in the country, and they were critical to the Covid-19 response [16].

Qualitative studies in South Africa on nurses and the Covid-19 pandemic have explored the lived experiences of professional nurses (with four years of training) in a public hospital [17] or in the intensive care units (ICUs) of a private hospital [6]. Nyandeni and colleagues found that professional nurses who nursed Covid-19 patients suffered psychological distress and burnout, exacerbated by insufficient resources and lack of managerial support, which in turn affected the quality of patient care [17]. Molala and Downing found that the abrupt transition from ‘normality’ to the pandemic, and isolation from family, community, and nursing management, were offset by their feelings of professional satisfaction and gratitude for teamwork and learning [6]. However, neither of these studies focused on ethical dilemmas and/or moral distress explicitly.

Although not comparable because of differences in methodology, surveys have explored the levels of and factors associated with psychological distress among healthcare workers in a public sector hospital during the first Covid-19 epidemic wave [18], the post-traumatic stress and coping strategies of nurses during the second epidemic wave [19], and the impact of the Covid-19 pandemic on nurse outcomes (e.g. compassion satisfaction, burnout, intention to leave) in the private sector of South Africa [20]. One survey among public sector healthcare workers found a high degree of psychological distress, influenced by perceived risks, training and supportive workplace relationships [18]. Another one found high levels of post-traumatic stress disorder, worsened by the unpreparedness to manage Covid-19 patients, poor personal health, and avoidant coping behaviours [19]. Among private sector nurses, sizeable proportions indicated their intention to leave their job or the profession worsened by increased exposure to death and dying [20]. However, none of the surveys focused on ethical dilemmas or moral distress. An integrative literature review underscored the paucity of studies on nurses’ moral distress in low-and-middle income countries (LMICs) [21]. Although Covid-19 was not the review focus, the authors recommended more qualitative studies among nurses in LMICs and in various cultural settings to address the knowledge gaps on moral distress [21].

This purpose of the study was to explore the experiences, specifically the ethical dilemmas and moral distress, of critical care nurses during the Covid-19 pandemic in a South African province, using Gilligan’s ethic of care theory [22] as the conceptual framework. The rationale for the study is both its scholarly contribution and its policy relevance. The study begins to address the knowledge gaps on South African nurses’ experiences of caring for patients during the Covid-19 pandemic in critical care units. The study contributes to the discourse on healthcare ethics, particularly in crisis situations, and the need for robust support systems for nurses, and has global relevance.

## Methods

### Conceptual framework

This paper draws on Carol Gilligan’s ethic of care theory [22, 23]. Originally developed as a feminist critique of traditional moral development theories, the ethic of care theory emphasises the importance of context, relationships, empathy, and care in ethical decision-making [23]. There are four central aspects of Gilligan’s Ethic of Care theory that informed this qualitative study. Firstly, the theory highlights the notion of *contextual morality*, namely that individuals consider the specific circumstances and relationships involved in ethical decision-making in [23]. In this study, the context was critical care units in a South African province. Secondly, the theory has a *relational focus* which means that moral reasoning is grounded in inter-personal relationships and the needs of others. In this study, critical care nurses relate to patients, their families, and with other members of the healthcare team. Thirdly, Gilligan’s theory underscores *empathy and compassion*, which implies that ethical decisions are made by considering how actions affect others both emotionally and socially [22]. Hence, critical care nurses consider how care provision might affect patients and their families. Lastly, Gilligan emphasises *voice and inclusion*, particularly the importance of listening to and documenting the experiences of those marginalised in traditional moral discourse [23], and in this study, the focus is on nurses who remain undervalued in health care systems. We used Gilligan’s ethic of care theory in the design of the interview schedule, the phrasing of the questions, the thematic analysis of the interviews with critical care nurses, and in the subsequent interpretation and discussion of the results.

### Study setting

The study setting was the Gauteng province of South Africa, home to more than one-quarter of South Africa’s population [24]. The province is the economic hub of the country, generating more than one-third of the gross domestic product. During the pandemic, the province accounted for 33.1% of the total reported Covid-19 cases, a cumulative total of 101,697 Covid-19 deaths, and an estimated 64 522 excess deaths [25, 26].

### Study design

This was an exploratory, descriptive qualitative (EDQ) study that sought to explore the experiences of critical care nurses during the Covid-19 pandemic. Hunter and colleagues note that the EDQ study design “*allows the researcher to explore a topic with limited coverage within the literature and allows the participants of the study to contribute to the development of new knowledge in that area*” [27]. The EDQ design was suitable because there was a dearth of information on the ethical dilemmas and moral distress of critical care nurses during the Covid-19 pandemic in South Africa. Hence, the study design enabled the research team to capture the voices of nurses, widely regarded as the “backbone” of South Africa’s healthcare system [19], yet often vilified for their attitudes to and relationships with patients [28].

### Participant recruitment and selection

The inclusion criteria for the study were nurses working in an adult critical care or intensive care units (ICU), experience of nursing individuals with Covid-19, and providing voluntary informed consent. We recruited nurses through professional associations and/or trade unions, key informants, placing an advert in LinkedIn, and snowballing. We used snowball sampling until there was no new information emerging from the interviews. Hence, this repetition signalled that data saturation was reached [29].

### Data collection tool

Following an extensive literature review on Covid-19, ethical dilemmas, moral distress, and using Gilligan’s ethic of care theory, the principal investigator (PI = BS) developed a semi-structured interview schedule in English. The semi-structured interview schedule was developed specifically for this study (Annexure 1). The interview focused on the personal experiences of the ICU nurses during the Covid-19 pandemic in Gauteng, their experiences of taking care of patients with Covid-19, their perspectives on ethical dilemmas faced during the pandemic, relationships with other colleagues and/or management, the availability of ethical guidelines and/or discussion forums, any support mechanisms e.g. training on Covid-19, the availability of PPE, and any other comments that they wished to make.

The principal investigator (BS) piloted the interview schedule with two critical care nurses to test the clarity of the questions, and the time taken for the interview. There was no need for revisions to the interview schedule. The pilot study findings are excluded.

### Data collection

Between June and October 2022, eligible nurses who responded to the recruitment drive were invited to participate in the interviews via email or telephone. Each nurse received a detailed information sheet that explained the voluntary nature of participation, as well as the ethical principles of confidentiality, and anonymity. The PI developed a detailed distress protocol to ensure that participants had support should they experience distress or discomfort during the interview. The information sheet also included information on the distress protocol and the contact details of psychological support services.

Once the nurse agreed to participate in the study, a mutually convenient date was set for the in- depth interview. After obtaining informed consent, the in-depth interviews were conducted with the nurses using the semi-structured interview schedule. All interviews with the nurses were conducted in English. All the Covid-19 protocols of mask wearing, hand hygiene and physical distancing were adhered to. The PI also took detailed field notes during the interviews.

Following each interview, the PI shared information about the Health Care Workers Care Network [30]. This was a unique Network in South Africa established to provide free confidential counselling sessions specifically for healthcare workers during the pandemic.

All interviews were recorded digitally with informed consent and labelled with a code. All audio-recordings are kept on a password-protected computer to ensure confidentiality.

### Data analysis

The audio recordings were transcribed verbatim. The PI (BS) selected three transcripts following quality checks. The PI, senior author (LR) and a retired professional nurse, researcher and educator, read the transcripts independently, using inductive coding [31]. The data was first mapped as a means of reviewing the selected transcripts and identifying information that stood out. Immersion in the data was undertaken through reading and rereading the transcripts along with the fieldnotes that were taken during the interviews. The notes included information on whether the nurse was from the private or public health sector in Gauteng, age, gender, and category.

Once the independent coding was complete, a meeting was held to discuss the independent codes and themes, and to reach inter-coder agreement. An iterative process followed of examining the inductive codes and themes in light of the study objectives, and Gilligan’s theory. Once the themes were developed, the PI analysed the remainder of the interviews. The PI and senior author held several follow-up meetings to refine the themes and sub-themes as guided by the data and Gilligan’s theory. Following peer-review, the subthemes were further consolidated based on data immersion, conceptual similarities, and the ethic of care theory.

### Rigour and trustworthiness

Rigour refers to the strategies used to ensure the quality, credibility, and reliability of the study [32]. In this study, rigour was ensured by having an explicit theoretical framework, an appropriate study design that incorporated suitable guiding questions and meticulous study execution.

Trustworthiness includes the criteria of credibility, dependability, confirmability, and transferability [32]. Three interviews were randomly sampled for intercoder agreement to ensure reliability [33]. The PI, supervisor and an independent researcher coded the same three transcripts independently to ensure credibility. All the transcripts were read carefully, and several meetings were held to discuss coding discrepancies until an agreement was reached. The PI ensured prolonged engagement with and immersion in the data. The supervision meetings with the senior author ensured regular discussion, debriefing, and reflection.

We used a semi-structured interview schedule and provided a detailed description of the methods, data collection and data analysis undertaken to ensure dependability [33]. Confirmability refers to whether the findings are shaped by the participants and not researcher bias [32]. The interviews were described verbatim from the voice recording and checked against the audio-recording. Confirmability was also ensured by taking detailed field notes during data collection, and this was supported by reflexivity and maintaining a clear audit trail of data and interpretations. The results use verbatim quotes to reflect the experiences of critical care nurses.

Transferability refers to the extent to which findings can be applied to other contexts [32]. We achieved this through a thick description of the research setting and participants so others can judge the applicability of the findings in their own settings.

### Researcher positionality

The PI has experience as a psycho-social counsellor in assisting individuals with anxiety, depression, and developing appropriate coping strategies. This experience was advantageous in conducting the interviews with critical care nurses during the pandemic. The PI adopted a listening and empathetic approach that allowed participants to share their experiences. This encouraged the nurses to elaborate on routine nursing care in an ICU, and how the Covid-19 pandemic influenced care provision, thus enriching the depth of the interviews. The PI documented the emotions, assumptions and experiences of doing the interviews in a journal.

The senior author (LR) is a qualified nurse and midwife with extensive experience of working in ICUs, and postgraduate degrees in epidemiology, economics and public health. LR is a professor of public health and holds a Research Chair on the Health Workforce.

Both authors are women of colour, committed to social justice and human rights. An important part of conducting the research study, was to ensure reflexivity [34] and how this might influence the research study. Data analysis was an iterative process, and combined with reflexivity, to ensure that any potential biases did not influence the findings.

## Results

### Demographic characteristics

Twenty-one (21) interviews were conducted with nurses working in ICUs in Gauteng province, comprising 16 females and 5 males. Most of the participants were black (20/21 = 95%) and working in a public hospital (17/21 = 81%). Twenty participants were professional or registered nurses (with at least four years of nursing training) and one participant was an enrolled nurse (with at least two years of nursing training).

### Emerging themes

Table 1 shows the five themes and sub-themes that emerged from the interviews, reflecting the ethical, emotional, and systemic dimensions of frontline nursing care in a time of crisis, namely the Covid-19 pandemic. The first theme, deep caring for patients versus pragmatic compromises, captures the tensions between nurse’s commitment to quality patient care and their inability to provide quality or dignified nursing care. The second theme, ambivalence about the Nursing Oath explores how the pandemic led some nurses to feel conflicted about the ideals embedded in their professional oath amidst the huge personal risk of Covid-19. The third theme, emotional labour, uncertainty and fear, illustrates the emotional labour and moral distress of managing very sick patients whose conditions deteriorated rapidly, dealing with large numbers of patient deaths, exacerbated by uncertainty and fear of the Covid-19 pandemic. The fourth theme, lack of appreciation and investment reflects the participant’s perceptions of being undervalued in the health system. The last theme, navigating resource constraints focused on health system limitations or inadequacies such as staff shortages, inadequate PPE and limited supplies, all which shaped nurses’ experiences during the pandemic. These systemic inadequacies exacerbated the ethical dilemmas and heightened the moral distress.

These five emerging themes are aligned with Gilligan’s ethic of care theory [22] and centre on both context, and the ethical dilemmas experienced by nurses in relation to patients (themes 1 to 3), and nurses and the health system (themes 4 and 5). Although the themes overlap, each is described separately for the sake of clarity.

**Table 1:** Emerging themes and subthemes

Theme	Subtheme	Relation to Gilligan’s theory
1. Deep caring for patients versus pragmatic compromises	• Cutting corners in nursing care provision • Absence of touch or inability to hold patients’ hands • Restrictions on visitation • Perceptions of dubious medical treatment • Inability to treat dead patients with dignity	• Relational focus • Empathy and compassion
2. Ambivalence about the Nursing Oath	• Nurse safety vs. prioritising patient care • Overtime obligation due to absent colleagues or insufficient staff • No choice whether to work	• Relational focus • inter-personal relationships • Needs of others • Empathy and compassion,
3. Emotional labour (moral distress), uncertainty and fear	• Rapid changes in condition of patients • Patients ‘dying like flies’ • Feelings of regret for patients who could not be saved • Loss and grief at the death of colleagues and family • Shock and fear of the Covid-19 pandemic • Knowledge gaps and changes in regulations • Conflict, stigma and isolation (colleagues, family, friends)	• Context (uncertainty, knowledge gaps, etc.) • Relational focus (inter-personal relationships, Needs of others) • Empathy and compassion,
4. Lack of investment and appreciation	• Perceived lack of value attached to health workers: exposure to Covid-19; put in the firing line • Insufficient training, information or guidelines • Lack of management visibility (no rounds from seniors) • Little acknowledgement or expressed appreciation	• Contextual morality • Listening and voices
5. Navigating resource constraints	• Insufficient number of beds or ventilators • Poor-quality PPE/inappropriate sizes of PPE • Human resource fragilities • Lack of supplies	• Contextual morality

### Theme 1: Deep caring for patients versus pragmatic compromises

This theme centres on the tension between the principles of deep caring for patients and the realities of the personal and ethical struggles faced by nurses during the Covid-19 pandemic. It comprises of five subthemes: cutting corners in nursing care provision; absence of touch or inability to hold patients’ hands; restrictions on visitations, perceptions of dubious medical treatment; and an inability to treat corpses with dignity.

The Covid-19 crisis necessitated pragmatic compromises in ICUs, which involved doing the bare minimum, spending limited time with each patient, and in some instances ‘cutting corners’ with turning patients or doing bed baths. One interviewee noted that:

*In ICU*,* patients cannot turn themselves*,* so bed baths are very beneficial to prevent bed sores. So*,* when you do a thorough bed bath you have to wash*,* you have to rub the patient. But now there was* [Covid] *and we are not sure if it is contact or air. So*,* we did the bare minimum.* (P5, Female, 24 years, Professional Nurse, Public Hospital).

Another participant highlighted that the situation necessitated shortcuts and said:

*In ICU you have to turn* [the patient] *four-hourly*,* and we did not do these turnings. We only did* [the turning] *if somebody has messed themselves up*,* that was when you know you cannot leave that patient for long. But if you did not*,* they would stay in that position almost 12 h. You know we were not ready* [for the pandemic], *we did not have the partitions where you separate patients. So*,* that also put a challenge on nursing because you know we are always short* [of nurses]. *So*,* if we have 11 patients*,* how will you get 11 nurses dealing with that? Somehow*,* we had to cut corners* (P10, Male, 42 years, Professional Nurse, Public Hospital).

The simple act of touch or handholding was a risk during the pandemic. Nurses spoke movingly of the distress at their inability to touch patients or hold their hands, exacerbated by the wearing of personal protective equipment (PPE). This is reflected in the poignancy of the following narrative:

…*the type of care that we give to patients*,* is important*,* I need to feel your pulse*,* I need to touch you. I cannot give you empathy and sympathy if I cannot show that to you. You need to see my face; you need to feel my touch. So*,* those were the barriers that Covid had created*,* because we had to wear this PPE*,* which clouded who you were. I could not give a smile under a mask. You could not see me under that whole PPE because I am donned in an attire that closed who I am*,* my head is closed*,* you cannot see my head*,* sometimes you cannot even see me*,* because we probably have got glasses on*,* I have got a mask*,* and I have got a visor. And I have got these long boots and these gloves and everything. You cannot tell whether I am black or white. You cannot relate with me…. it was impossible”.* (P8, Male, 33 years, Professional Nurse, Public Hospital).

The visitation restrictions also created ethical challenges as families were not allowed into hospital or critical care units due to the risk of exposure and infection transmission. The nurses highlighted that part of the healing process requires family support for the patient but due to patient isolation and no visitation regulations, patients were alone without the much-needed support. The lack of family support added to the distress experienced by nurses. Some nurses expressed deep sadness at the time when some patients would die without seeing a family member.

*They* [family] *couldn’t even come and just sit and support them* [patients], *it was so painful* (P3, Female, 30 years, Professional Nurse, Public Hospital).

In some instances, the nurses went out of their way to ensure that families were kept informed of the patient’s condition.

*I must say*,* the family were just so thankful that they could get any information. We would try to call them*,* the nursing staff would take their own phones*,* and we would call them via WhatsApp and do a video call with families*,* or anything because they couldn’t come in. It was bad for us* [the nurses] *that the families couldn’t be there. By the second wave* [of Covid-19 infections], *if we saw that a patient was going to die*,* we said; no*,* they* [the family] *must come*,* you can’t let a patient die alone*,* it is just too traumatic. It was traumatic for everyone. So*,* we allowed them*,* we would dress them up and let them come in* (P4, Female, 42 years, Professional Nurse, Private Hospital).

The Covid-19 pandemic was an unknown, and in the early stages evidence-based treatment protocols were lacking. The nurses questioned the efficacy of medical treatment, and found the trial and error approach challenging.

*Like I said it was trial and error for everyone*,* so even the medications that were not working*,* we would just give you*,* the antibiotics and whatever*,* we were just giving* (P14, Female, 46 years, Professional Nurse, Public Hospital).

In some instances, the experience of witnessing a patient’s demise left the nurses with feelings of regret and the consideration that under normal care circumstances some patients would not have died. They also expressed feelings of failing patients during Covid-19.

Nurses reported that the hospital mortuaries were overwhelmed and sometimes there were delays in receiving the corpses. There were no guidelines on how to manage the deceased with dignity. Furthermore, there was no space to stow the bodies that were awaiting the mortuary.

*You know as a nurse*,* you nurse until end of life*,* you do the last offices even the dignity and then the moral aspect of it*,* like you take every patient as if it was my sister if this was my mother*,* but with Covid we went through the most. When we found piles and piles of corpses*,* we did not know how to handle them*,* the fridges were full*,* like the dignity between that part of last offices was lost altogether*. *They were just treated like… I don’t know because the death*,* the mortality rate was so high they were dying at an accelerated rate*,* up there was nowhere to put them*,* how to treat the corpses with dignity* (P3, Female, 30 years, Professional Nurse, Public Hospital).

*It was worse because we saw people dying every day*,* I am not even going to say almost every day because it would be sometimes three corpses per shift. And unfortunately*,* we knew when we had an admission with that person that they would not return home.* (P16, Female, 43 years, Professional Nurse, Public Hospital).

As ICU beds were in high demand, after a patient’s demise, another patient would need to use that bed in the shortest time possible resulting in corpses piling, which resulted in further distress for nurses.

*We were just dealing with a number. Even when you came in*,* they were like ‘we have five corpses*,* we have ten more admissions’*,* so it was like a push out*,* push in push out*,* push in*. (P3, Female, 30 years, Professional Nurse, Public Hospital).

*It was bad*,* you know*,* it was very bad. You want to admit the other* [patient] *sitting on the chair. The bed is still occupied by the* [dead] *one who needs to go to the mortuary*,* it was very bad.* (P1, Female, 53 years, Professional Nurse, Private Hospital).

### Theme 2: Ambivalence about the nursing oath

This theme highlights the ambivalence expressed about the Nursing Oath in the context of Covid-19. The three sub-themes reflect the ethical dilemma of their own personal safety as nurses versus prioritising patient care; overtime obligations due to absent colleagues or insufficient staff; and little or no choice about work.

Nurses were acutely aware of the moral obligation to put the health of their patients as their first consideration and to provide compassionate and dignified nursing care. However, nurses had to balance their obligations with the personal risk of infections and disease exposure. This tension is highlighted by the following response.

*The dilemma was that in the* [Nursing] *Oath that we take*,* there are lines that you will put the needs of your patient before your personal needs. And there is no prejudice in how you offer the service. So*,* whatever condition and whatever state of health you* [the patient] *come in*,* I am responsible for giving you the necessary service. Now*,* how do I then put myself in the same state*,* or in the same fashion as I would for anyone with any other condition*,* when now I know this person* [with Covid-19] *poses a 100% threat to my life?* (P8, Male, 33 years, Professional Nurse, Public Hospital).

At times they would find themselves with the dilemma of putting on the PPE that would take around five minutes to don or whether to prioritise a critically ill patient where every minute counted.

*You know when you are working in an ICU*,* that person is crashing* [deteriorating quickly] *or when you have an emergency you have to attend to that person immediately. With the Covid-19*,* you have to protect yourself first before you nurse that person meaning you have to wear all these things* [PPE]. *By the time you get in*,* it’s too late.* (P16, Female, 46 years, Professional Nurse, Public Hospital).

The nurses interviewed highlighted the difficulties in decision-making, which created various ethical dilemmas. At times, they felt selfish when they thought about their own wellbeing first instead of the patient as directed by the Nursing Oath. They also felt conflicted by the messages from trade unions which encouraged them to put their own safety first.

*The unions and the other people that were in charge of Covid management will tell you that there is no emergency in Covid because you have to protect yourself first before you can rush* [into the Covid-19 ward or ICU] (P9, Male, 28 years, Professional Nurse, Public Hospital).

Nurses also expressed their feelings of ambivalence of witnessing patients being prematurely and unjustly written off by doctors, when they felt that more could have been done in line with the principles of the Nursing Oath. The nurses interviewed said that they felt compelled to work in the Covid-19 wards, which was daunting. In some cases, they thought that the Nursing Oath was ‘imprisoning’ them and forcing them to do what they did not want to do. When assigned to work in the Covid-19 ward, it felt like a dead sentence.

*We were imprisoned* [to work in the ward], *obviously*,* the* [Nursing] *Oath*,* as well as the contractual obligation that we signed with the Department* [of Health] (P8, Male, 33 years, Professional Nurse, Public Hospital).

*We were forced to go in because*,* it is a catch-22. You care for* [patients], *you look at them suffering and then you cannot just let them suffer. You so just make do with whatever you have*,* go and attend to the patient and then come out again* (P6, Female, 34 years, Professional Nurse, Public Hospital).

### Theme 3: Emotional labour, uncertainty and fear

This theme captures the nurses’ narratives that highlight the emotional labour, the uncertainty, and the fear of taking care of patients during the Covid-19 pandemic. Seven subthemes capture the depth and complexity of their ICU nursing experiences: rapid changes in the condition of patients, patients ‘dying like flies’, feelings of regret for patients who could not be saved, loss and grief at the death of colleagues and family, shock and fear of the Covid-19 pandemic, knowledge gaps and changes in regulations; and conflict, stigma and isolation (colleagues, family, friends). The expressed fears intersected and included fear of the pandemic, of infection, of the unknown, and of being in the frontline of health care provision.

Nurses expressed feelings of helplessness due to the large number of patients that were admitted in hospitals and the limited capacity at the hospitals:

*I think the fact that we felt we wanted to treat more people*,* but we couldn’t. Especially the second and the third waves were very severe*,* we just couldn’t take more* [patients], *the ambulances were standing outside with patients* (P4, Female, 42 years, Professional Nurse, Private Hospital).

They were greatly stressed by patients who initially appeared stable, but would deteriorate rapidly, and subsequently pass away. There was a general perception that “patients were dying like flies”.

*One minute you are talking to her*,* the next minute she is hyperventilating and then she’s anxious and then she’s getting intubated and then she is dying! Tomorrow when you come*,* she is no more. It was just a bad experience seeing people … people were dying like flies* (P1, Female, 53 years, Professional Nurse, Private Hospital).

*Within three hours*,* that person is intubated*,* within a few days they pass away. So*,* they* [patients] *would come* [into ICU] *talking and you will just see them really deteriorating and eventually passing on* (P14, Female, 46 years, Professional Nurse, Public Hospital).

*In fact*,* I cannot say we were there to save lives; we were just there to help create beds. You know if you get healed then go out*,* good for you. But most of the time*,* especially the first*,* second*,* and third* [infection waves], *the second was a bit minimal*,* it was not that bad*,* but the first and the third it was all about me being there to help you die in dignity. We were helpless* (P14, Female, 46 years, Professional Nurse, Private Hospital).

The context of different lock-down stages and the government’s changing Covid-19 regulations intersected with the nurses’ fears. Their narratives reveal how scary the pandemic felt, the difficulties of dealing with constant changes, and of interpreting these changes for nursing care. Nurses highlighted the complexity of taking care of patients with Covid-19, because their progress and responses to treatment were very different.

Simultaneously, critical care nurses also had to deal with the grief of losing their colleagues, family and friends

*I lost a colleague*,* and I lost a sister*,* so I am feeling like they were also neglected just as much as other patients were neglected. Some of the patients were not even supposed to die* (P7, Female, 32 years, Professional Nurse, Public Hospital).

*We were losing colleagues; we were losing family members. And I think another very depressing thing was that you could not even go and bury people because of the travelling bans. We were losing colleagues in the same hospital that they used to* [work in]. *I remember we had one ICU nurse. She got sick and she said she wanted to be nursed in* [this] *ICU*,* and she passed on* (P5, Female, 24 years, Professional Nurse, Public Hospital).

Nurses talked about the negative atmosphere in the hospital caused by the loss of family and friends, which made it seem as though the hospital was a place of death, rather than recovery. In some instances, nurses advised their family members to treat themselves if they tested positive and to avoid hospitals. They highlighted their perceptions of the hospital as an unsafe place that presented a moral hazard to their wellbeing. They also felt stigmatised for working in the Covid-19 ward.

*“If they* [fellow colleagues] *find me using the phone*, [they would] *wipe the phone and wipe the desk where I was sitting. If I go and have tea in the morning*,* they just leave the room* (P19, Female, 32 years, Registered Nurse, Private Hospital).

These fears were exacerbated by the closeness of the pandemic, and the nature of nursing which involves close contact with patients, illustrated by the comments from three nurses interviewed.

*It* [Covid-19] *was like a bullet that I could not dodge. Because at some point there were patients where the initial test was negative*,* they would be in the unit. But then they would get complications and then they would retest and then it comes back positive* (P6, Female, 34 years, Professional Nurse, Public Hospital).

*It was scary because you thought maybe the air that I’m breathing*,* the Covid is coming*,* like getting to work you don’t even trust doctors. Anybody who is next to you maybe they are having Covid*,* you know*,* it was very scary*,* and we were not used to the methods and protocols and everything* (P1, Female, 53 years, Professional Nurse, Private Hospital).

*When we first heard about it*,* it seemed very far away; the whole thing about it starting in China*,* and then you could feel it coming closer and closer. I think we all knew about Italy and then the US*,* and you could feel it coming closer* (P4, Female, 42 years, Professional Nurse, Private Hospital).

Nurses’ fears were also made worse by the lack of or minimal information about the virus and disease, as well as lack of clarity on the expectations from health workers.

*Nobody knew exactly how Covid was being transmitted*,* so it was very difficult to manage* [and] *know how to treat patients* (P2, Female, 51 years, Professional Nurse, Public Hospital).

*It was a very scary experience because we did not know what was expected of us* (P3, Female, 30 years, Professional Nurse, Public Hospital).

In the context of limited information on nursing care for patients with Covid-19 nurses often relied on Google or fellow colleagues for relevant information. In some instances, doctors shared information with the nurses. The lack of information intersected with the urgency of the pandemic and contributed to the pragmatic compromises, such as taking the decision to withhold information about the procedures undertaken, because they did not want to scare the patients.

*Whatever you are doing*,* you need to inform the patient. But*,* in some cases*,* I was compelled not to utter a word for fear of scaring them*,* I just opted to do what was best for them* [the patients] (P2, Female, 51 years, Professional Nurse, Public Hospital).

Those nurses that used public transport to commute to work experienced stigmatisation because they were working in a hospital:

*During Covid*,* you could not get into a* [mini-bus] *taxi as a nurse. And most of us were not driving at that time. Even besides the stigma*,* you yourself are thinking*,* ‘I was nursing Covid-19* [positive patients] *and now I could be contaminating many people* [in a taxi] (P12, Male, 33 years, Professional Nurse, Public Hospital).

*Immediately you walk in a shop*,* everyone just goes out*,* you walk into a queue*,* people start walking away. You are in a taxi rank; people do not want to sit next to you and all those things* (P19, Female, 32 years, Registered Nurse, Private Hospital).

In some cases, nurses experienced isolation from family and friends. The high-pressure ICU environment also resulted in tense relationships and, in some instances, conflict, amongst hospital staff. Nurses reported that some supporting departments refrained from coming to the Covid-19 wards:

*So*,* other departments*,* for example physiotherapy*,* were not willing to attend to the patients* (P5, Female, 24 years, Professional Nurse, Public Hospital).

*Even with the doctors you will call a doctor for 2 h. Sometimes they’ll come when the patient is dead*,* so even for them* [the doctors] *I do not want to lie*,* relationships were not good at all because we will end up exchanging words* (P7, Female, 32 years, Professional Nurse, Public Hospital).

Some nurses talked about their experience of fatigue and burnout, because their colleagues contracted Covid-19 and were on sick leave, leaving the remaining staff to take care of critically ill patients.

### Theme 4: Lack of appreciation and investment

This theme focuses on nurses’ perspectives of the lack of appreciation for their work, for risking their lives as health care providers, and the lack of or under-investment in their personal and emotional well-being. it consists of four subthemes: perceived lack of value attached to health workers, insufficient training, information or guidelines, lack of management visibility (no rounds from seniors) and little acknowledgement or expressed appreciation.

Nurses believed the most visible illustration of the lack of appreciation was the failure of government to give public servants the annual cost of living increase, as well as the lack of financial incentives.

*Yes*,* South Africa was managing Covid. Who was managing Covid? It was us*,* the nurses. But we were never appreciated. They* [government] *cut our increment* [annual increase]. *We never had Covid incentives. Nothing. We had nothing* (P13, Female, 48 years, Registered Nurse, Public Hospital).

*And the worse constraint is working in an environment that is life-threatening*,* but you are not financially well taken care of. So*,* in terms of the salaries*, [these] *never increased*. (P15, Female, 34 years, Professional Nurse, Public Hospital).

Nurses reported that they were promised the opportunity to claim reimbursement for the extra hours and commitment during the pandemic, especially when they were sick and decided to come to work for the benefit of patients. The majority of the nurses interviewed complained about the lack of incentives received as expected, leaving them feeling ‘*used and not valued****’***.

In the private hospitals, there appeared to be some flexibility, with hospitals offering an allowance to nurses if they took care of patients with Covid-19.

*Everyone was terrified*,* everybody confused. To an extent whereby the hospital was saying*,* “We will pay*,* if you nurse a Covid patient then you get this specific allowance”. So*,* then people started to take care of Covid patients but then it faded away. Somehow that is good in a way because it is an incentive for risking your life.* (P17, Male, 30 years, Professional Nurse, Private Hospital).

Some nurses highlighted the lack of support from their managers during the pandemic, some who were reportedly too scared to visit the ICU. One nurse said:

*They* [management]*were a no show; they will only call when they need a report. They did not even want to see where you’ve written your report* (P7, Female, 32 years, Professional Nurse, Public Hospital).

In the early stages of the pandemic, the problems with PPE were exacerbated by the lack of training on infection prevention and control, as well as the lack of clear guidelines on Covid-19. As the pandemic progressed there were more training opportunities as well as the use of master trainers in institutions:

*Yes*,* we did. We did a lot* [of training]. *Especially in terms of contamination*,* I have three certificates. And we were given links for online training as well. With the second wave. I remember I went for Covid vaccine training* (P5, Female, 24 years, Professional Nurse, Public Hospital).

*There were people* [fellow colleagues] *who were elected to be trained and then they would come back to us and do that debriefing here.* (P10, Male, 42 years, Professional Nurse, Public Hospital).

### Theme 5: Navigating resource constraints

This theme highlights the ethical dilemmas associated with nurses having to navigate resource constraints and the impact on nurses’ ability to provide quality care during the pandemic. Participants described working in environments where there were insufficient numbers of beds and ventilators, a lack of adequate treatment options, and poor-quality or ill-fitting personal protective equipment (PPE). These constraints are captured under the subthemes of insufficient number of beds or ventilators, poor-quality PPE/inappropriate sizes of PPE, human resource fragilities and lack of supplies.

The lack of PPE, insufficient or poor-quality PPE, especially in the public sector, was a constant struggle for nurses, and this added to their stress and moral distress. One nurse highlighted this distressing experience in a powerful narrative:

*We were like soldiers*,* we went to a war where there were bombshells*,* where there was firing*,* and you knew for sure*,* the moment someone is firing back at you. And you had 100% chance of the bullet hitting you*,* and that is exactly what was happening with Covid. Because we did not have enough PPE. We did not even have quality PPE*,* even the PPE we had* (P8, Male, 33 years, Professional Nurse, Public Hospital).

In other instances, when the PPE was available, there would be challenges with the quality or the sizes to the point where being assigned to work in the Covid-19 ward was determined based nurses who could fit into the small sizes.

*The sizes were challenging*,* because they were small sizes most of the time. So*,* there were certain people that would fit in*,* you have to do even the juggling of nurses. The big ones would not go in *[the Covid-19 ward], *because they do not have proper PPE.* (P10, Female, 42 years, Professional Nurse, Public Hospital)

Notwithstanding the problems with PPE, many nurses reported that they improvised and found ways to provide care for sick patients.

*We donned* [plastic] *garbage bags instead of the proper PPE gowns. And we would go in* [the Covid-19 unit] *and work and then come back out* (P6, Female, 34 years, Professional Nurse, Public Hospital).

*So even suctioning a patient who is Covid-19 [positive] that time it was a scare even just a droplet coming to your face… Because we were the first ones to go to the Covid unit that they opened. We were using the* [plastic] *aprons*,* even* [ordinary] *plastics because I mean we are even trying to wrap our feet*,* our faces*,* our hair like everything that we could get our hands on. We even brought our clothes from home*,* that we knew that we’re not going to use anymore* (P7, Female, 32 years, Professional Nurse, Public Hospital).

In some instances, nurses purchased masks at their own expense to ensure they could perform their duties safely.

*Literally* [I would] *go to Dis-Chem *[a private pharmacy],* buy a box* [of masks] *and keep it in your car or whatever it is and make sure you carry a few masks for yourself in your bag* (P11, Female, 28 years, Professional Nurse, Public and Private Hospital).

In contrast to the experience of their colleagues in the public health sector, private sector nurses reported that there was sufficient PPE.

*I heard other nurses didn’t* [have PPE] *but where I was working it was enough. I don’t remember working without a scrub* (P1, Female, 53 years, Professional Nurse, Private Hospital).

The increased number of patients at hospitals resulted in insufficient beds available, lack or insufficient ventilators at some hospitals, and the shortages of supplies.

*There was a shortage of masks*,* there was a shortage of gowns*,* you had to repeat your mask for maybe three to four days.* (P12, Male, 33 years, Professional Nurse, Public Hospital).

*We had limited resources and in terms of beds available for sick patients coming in*,* at some point there had to be changes. We were not ready for such a disaster; you would find patients put in spaces where they were not properly isolated from other patients.* (P15, Female, 34 years, Professional Nurse, Public Hospital).

Nurses highlighted the pre-existing shortages of critical care nurses before the pandemic, which were exacerbated by Covid-19, absenteeism of nurses who were quarantined because of contracting Covid-19, fear of coming to work and others that resigned from the frontlines. This meant that some nurses had to take care of two or three patients with Covid-19.

*We already had an ailing healthcare system*,* ailing*,* failing already in our known clinical problems*,* or conditions that we were fighting with*,* because we always had shortages of staff* (P8, Male, 33 years, Professional Nurse, Public Hospital).

*And there was a shortage*,* we lost a lot of nurses as well and some nurses just decided to stop* [working], *especially those who were already old*,* and having comorbidities*. (P14, Female, 46 years, Professional Nurse, Public Hospital)

*Due to staff shortages*,* we would end up actually contaminating* [Covid] *negative patients* (P5, Female, 24 years, Professional Nurse, Public Hospital).

The ICU nurses interviewed often had to deal with the dilemma of working in a high-pressure environment, the opportunity to get additional staff through nursing agencies who did not have ICU training and experience, providing care to seriously ill patients, and orientating and supervising these agency nurses. This added to their workload, and created more ethical dilemmas and further moral distress as they knew that inexperienced nurses should not nurse patients on ventilators.

## Discussion

The novelty of this qualitative study is the use of Gilligan’s feminist ethic of care theory to explore the ethical dilemmas and moral distress of critical care nurses during the Covid-19 pandemic in a South African province. This theory emphasises the importance of context, relationships, empathy, and the moral significance of caring for others [22].

The five themes that emerged from the nurses’ narratives highlight the complex interplay between their professional responsibilities and personal and emotional well-being. Many of the study participants likened their experience during the Covid-19 pandemic to that of a war situation, unable “*to dodge the bullet*” or having a “*100% chance of the bullet hitting*”, illustrating their perceptions of the continuous and unavoidable stress that they faced.

The nurses expressed a deep sense of caring for their patients, which aligns with Gilligan’s emphasis on empathy and relational ethics [22]. Sadly, this sense of ‘deep caring’ was at odds with the pragmatic compromises they had to make (such as limited patient turning or bed baths) due to the overwhelming demands of the Covid-19 pandemic. The nurses’ inability to touch and hold patients’ hands, coupled with hospital rules that prevented family contact or visits added to their moral distress. However, the moral distress was compounded by the rapidly worsening conditions of patients, watching patients ‘dying like flies’, overwhelmed hospital mortuaries, and their inability to treat the dead with dignity, thus creating feelings of neglecting and/or failing their patients. The study by Nyandeni and colleagues in a South African public hospital also found that nurses did their best to provide quality patient care, but patient deaths affected them negatively [17]. Similarly, Molala and Downing’s qualitative study among nurses in private hospital ICUs in South Africa underscored the moral distress that nurses experienced because of the high number of patients deaths and the loneliness of patients due to the visitor restrictions during Covid-19 [6]. Notwithstanding differences in context, qualitative studies on moral distress during the pandemic in Australia [35], and Taiwan [36] underscored the moral distress of caring for patients amidst a rapidly changing work environment, while an integrative review reported the moral distress experienced by nurses because they felt unable to provide the necessary care or support to patients [9].

Gilligan’s theory [22] suggests that moral distress is the conflict that arises because of the moral obligation to prioritise patient health and care amidst the uncertainty and fear of infection, the responsibilities of being on the frontline of service delivery, and the resource constraints and a perceived lack of appreciation from managers and health sector leaders. The study participants expressed ambivalence about the Nursing Oath or the South African Nurses Pledge of Service that guides the ethical and professional conduct of nurses [37, 38], reflects a deeper ethical conflict. Gilligan’s theory suggests that care ethics prioritise the well-being of others [22], but the Covid-19 pandemic forced nurses to balance patient care with their own personal safety. This expressed ambivalence underscores the complexity of care ethics in crisis situations, where the duty to care for others must be weighed against the need to protect oneself. The studies by Mulaudzi and colleagues in South Africa and by Sperling in Israel pointed out that nurses’ fears were complicated by the added risk and dilemma of infection transmission to their families, and/or friends, with potentially lethal consequences [39, 40].

In this study, those nurses in the public health sector spoke movingly about their perceptions of the lack of appreciation for their efforts and hard work, reflected in the lack of the annual cost of living increase for public sector staff, and the failure by government to pay the promised bonuses. The expressed need for compensation and recognition for the risks and hardships nurses endured during the pandemic was also found in qualitative studies in South Korea [41] and in Taiwan [42].

In our study, nurses highlighted their preference for visible support from nursing and/or hospital management to help them to navigate the challenges they faced. They wanted clearer information about the coronavirus, training in the ethical dilemmas and reassurance that management was behind them. This was also found in a qualitative study conducted in a public sector hospital in South Africa [17], in Finland and in a multi-country research study involving four African countries [43, 44], while a study among ICU nurses in Australia found that effective management communication strengthened nurses’ resolve to provide quality patient care during the pandemic [45].

Gilligan’s ethic of care emphasises the importance of context, and of recognising and valuing the contributions of caregivers in order to enable the relational and empathetic aspects of care [22]. The nurses perceived lack of appreciation and the resource constraints, especially the lack of PPE, contributed to the ethical dilemmas and moral distress experienced by nurses. Our study participants highlighted that the additional strain placed on limited resources in the public sector by the Covid-19 pandemic had a ripple effect on the overall quality of care provided to patients, and on their wellbeing as service providers. The ethical dilemmas made their daily work difficult and also led to moral distress. A 2021 study in Gauteng province that focuses on the health system response to Covid-19 during the first epidemic wave found that the pandemic exposed and amplified the health system fragilities [14]. The provincial government response failed to consider or deal with the fears of frontline healthcare workers, with no formal psychosocial support programmes in the early stage of the pandemic [14]. Similarly, a 2022 integrative review found that the ethical dilemmas experienced by nurses during the pandemic were caused by the lack of PPE, staff shortages, insufficient medical supplies and the uncertainties [9].

Nurses experienced several ethical dilemmas throughout the pandemic, many of which were shaped by the tension between their professional responsibilities and the difficult realities within the healthcare system. A recurring concern was the challenge of prioritising patient care while navigating personal risk, often in the absence of adequate protective equipment and clear guidance. The expectation to continue working despite illness or fear further limited nurses’ sense of autonomy and choice. Feelings of moral distress were compounded by systemic constraints, including staff shortages and limited resources, which compromised their ability to provide the level of care they believed patients deserved. Navigating conflicting guidelines and strained relationships with colleagues added to the emotional complexity of their roles. Collectively, these ethical dilemmas highlight the need for strengthened institutional support, ethical preparedness, and present leadership during times of crisis.

### Limitations and strengths

The interviews were conducted between June and October 2021, towards the end of the Covid-19 pandemic. A potential limitation is recall bias. However, the study participants are unlikely to forget their experience of nursing patients and/or their intense emotions during the pandemic. Social desirability bias is also a potential limitation when conducting face-to-face interviews. This was minimised by arranging the interviews at a time and place convenient for the nurses, the careful structuring of the questions, by putting the study participants at ease during the interviewing process, and by sharing a clear distress protocol. The study involved 21 nurses in one South African province, which means that the experiences of ICU nurses in other eight provinces could be very different. Lastly, the study only captured the perspectives of nurses who volunteered or responded to the advert. Their perspectives could be very different from other ICU nurses who chose not to participate in the qualitative study.

This study has numerous strengths. Firstly, this is one of the first qualitative studies to use Gilligan’s feminist ethic of care theory to explore the ethical dilemmas and moral distress of ICU nurses in an African country. We obtained rich narratives on the experiences of ICU nurses during a health crisis, and how these intersected or were made worse by lack of management support and resource constraints. Our paper adds to the discourse on the notion of a resilient health system that places frontline healthcare providers at its centre, able to withstand infectious disease outbreaks, ensure quality of patient care, and positive practice environments.

Lastly, nurses appreciated the distress protocol, which contained the contact details of free counselling services.

### Recommendations

The rich insights shared by the nurses highlight their dedication to patient care, the feelings of helplessness, the challenging situations they faced, and the need for management support.

There are several recommendations that arise from this study. The study highlights the urgent need for greater investment in the health and well-being of nurses, including psychological debriefing after traumatic situations, and dedicated psychosocial support services. There is strong evidence globally that the mental health of all healthcare workers was adversely affected by the pandemic [46, 47]. South Africa has occupational health and safety legislation which prioritises the safety of workers [48]. Mental health and psychological support programmes should be central to the implementation of this legislation.

The study highlights the importance of positive practice environments, the central components are safe work environments, availability of resources (e.g. equipment, medicines) to deliver quality care, professional recognition and empowerment, supportive management practices, and education and information that ensure that health professionals have the opportunities to learn and save lives [49]. Such environments could be achieved by raising awareness among managers or supervisors of the ethical dilemmas and moral distress experienced by nurses in ICUs. Importantly, the study highlights the importance of having regular and discussions and training on ethics and moral conduct in healthcare settings. One possibility is to workshop and brainstorm various scenarios, such as ethical conduct and patient care during crisis situations, including pandemics, climate shocks, and conflict or wars.

Lastly, the nurses expressed the desire for visible management support, which could have included their visits to the ICUs to speak to or engage with the frontline nurses, rather than avoiding them. We recommend improved communication between managers or supervisors, and nurses, which is likely to foster supportive workplaces.

## Conclusions

Gilligan’s ethic of care theory provides a valuable lens through which to explore the ethical dilemmas and moral distress of nurses during the Covid-19 pandemic. The theory highlights the ethical tensions and moral distress that arise when the ideals of care and empathy are challenged by a health crisis, personal risks, resource constraints and indifferent managers and health care authorities.

The findings presented here offer insight into the complex reality’s nurses faced during the Covid-19 pandemic, shedding light on the systemic, emotional, and ethical challenges embedded in their daily work. The study contributes to the discourse on healthcare ethics, particularly in crisis situations, and highlights the need for robust support systems for nurses.

## Electronic supplementary material

Below is the link to the electronic supplementary material.

Supplementary Material 1

## Data Availability

The data and materials of this study are available from the corresponding author upon reasonable request.

## Abbreviations

Covid-19: Coronavirus disease of 2019
ICU: Intensive Care Units
PPE: Personal Protective Equipment
PI: Principal Investigator
