# Supplementary material for: ‘Unable to dodge the bullet’: a qualitative study of ethical dilemmas and moral distress of critical care nurses during the Covid-19 pandemic in a South African Province
Source: BMC Nurs. 2025 Jul 1;24:715. doi: 10.1186/s12912-025-03405-1 (PMC12211416; doi:10.1186/s12912-025-03405-1)
Supplement: Supplementary file 1 — Supplementary Material 1 [file 12912_2025_3405_MOESM1_ESM.docx]

**ANNEXURE 1**

**NURSES’ ETHICAL DILEMMAS AND MORAL DISTRESS DURING THE COVID-19 PANDEMIC IN THE CENTRAL HOSPITALS OF GAUTENG PROVINCE, SOUTH AFRICA**

**Semi-structured interview guide**

| Participant Code |  |
| --- | --- |
| Age |  |
| Gender |  |
| Occupation: Professional Nurse, Enrolled Nurse, Nursing Assistant or Other (specify) |  |
| Date of interview |  |

**INTRODUCTION**

1. Could you tell me about your experiences as a nurse during the COVID-19 pandemic?
   1. Probe: What were your initial thoughts when you heard that the first COVID-19 case was identified in South Africa in March 2020?
   2. Do you remember where you were?
   3. Would you mind sharing with me how you felt during the early stages of the pandemic?
   4. Did those feelings change later on?

**ETHICAL ISSUES**

1. What would you say are the ethical issues that you faced as a frontline nurse during COVID-19?
   1. Probe: Thoughts about the Code of Ethics for Nurses?
2. Tell me about your experience of taking care of patients with COVID-19 in the hospital where you are working? Probe:
   1. Patients: numbers, medical conditions, patient rights and conflict with their family members
   2. Did you experience a situation where you were unable to provide the care you would have wanted to provide a COVID-19 positive patient? When did this happen, during which wave? How did you deal with it?
   3. Health professionals: Skills and competence, interpersonal relations or collaborations, values and attitudes and own family and relationships
   4. Health system: perceptions of inappropriate care, resource constraints, PPE, and potential conflict with managers

**RELATIONSHIPS AND SUPPORT**

1. Could you tell me about your relationship with other colleagues?
   1. Probe: doctors, other nurses, other health workers?
2. What about your relationship with hospital management?
   1. Probe the unit manager?
   2. The operational manager?
   3. The Nursing service manager or CEO?
3. What support did you get in the ICU or hospital?
   1. Probe: training on COVID-19
   2. Availability of PPE?
   3. Other types of support (e.g., time off)
4. Were there discussions about ethical dilemmas or issues in the ICU or the hospital?
   1. Probe ethical guidelines?
   2. Ethical forums?
5. Were you faced with the situation of having to separate from your own family due to working with patients with COVID-19?
   1. Probe: quarantine
   2. Overall experiences
6. How would you describe your coping strategies during COVID-19?
7. Are there other comments you want to make about COVID-19, ethical dilemmas and/or support?
